# Physical activity and exercise for older people living with HIV: a protocol for a scoping review

**DOI:** 10.1186/s13643-020-01327-4

**Published:** 2020-03-20

**Authors:** Levin Chetty, Saul Cobbing, Verusia Chetty

**Affiliations:** Discipline of Physiotherapy, School of Health Sciences, University of KwaZulu-Natal, Westville Campus, Private Bag X54001, Durban, 4000 South Africa

**Keywords:** HIV, Geriatrics, Physical activity, Exercise, Rehabilitation

## Abstract

**Background:**

Older people living with HIV (OPLWH) are expected to live longer in the era of antiretroviral treatment, but at the same time, they are at risk for developing various health complications as a consequence of a life with the infection, exposure to medications that carry their own toxicity and side effects, and the natural effects of aging on the immune system. Because senescence is an inherent process that can be accelerated by HIV, it is important to identify strategies that can modify this phenomenon. Emerging data suggests that while physical activity and exercise may not have a positive impact on viral replication and on the immune system of people living with HIV, it can elicit improvements in cardiorespiratory fitness, strength, body composition, and overall quality of life. The purpose of this study is to map out empirical evidence on the effects of physical activity and exercise in OPLWH.

**Methods:**

The scoping review methods will be guided by the framework proposed by the Joanna Briggs Institute guidelines. Literature searches will be conducted in the following electronic databases (from inception onwards): PubMed/MEDLINE, Cochrane Library, and Google Scholar. Peer-reviewed journal papers will be included if they are written in English, involved human participants aged 50 years, and older with HIV and described a measure for physical, mental, or functional status of physical activity/exercise and/or the recommendations in OPLWH. Quantitative, qualitative, and mixed-method studies will be included in order to consider different aspects of measuring the effects of physical activity and exercise (e.g., quality of life, functional status, activities of daily living). Two reviewers will screen all citations and full-text articles. We will abstract data, organize them into themes and sub-themes, summarize them, and report the results using a narrative synthesis. The study methodological quality (or bias) will be appraised using a Mixed Methods Appraisal Tool.

**Discussion:**

The evidence gathered from the selected studies will be discussed in relation to the research questions using a narrative to identify and explore emergent themes. The review will provide a baseline of evidence on exercise and physical activity interventions for OPLWH. It will highlight gaps regarding the use of exercise and physical activity and contribute to the design of an effective intervention approach to the rehabilitation of OPLWH.

**Systematic review registration:**

Open Science Framework (https://osf.io/728kp/).

## Background

Older people living with HIV (OPLWH) are faced with various challenges that continue to influence their physical, mental, and societal roles within their communities. Globally, the number of people living with HIV (PLHIV) who are 50 years and older is predicted to increase by 47% to 6.9 million by the year 2020 [1]. Although there has been a recent surge in research around HIV and aging in developed or high-income countries, little is known about the intersection of HIV and aging in lower- to middle-income countries, especially sub-Saharan Africa, which accounts for 62% of newly diagnosed HIV infections among people who are 50 years and older [2].

ART has increased the longevity of PLHIV, but at the same time, it is associated with a constellation of health problems and physical impairments such as sarcopenia and peripheral neuropathies, which can greatly affect functional activities [3]. Functional limitations and disabilities within this population are just not due to the disease itself but also the secondary effects of ART [4].

As life expectancy inclines steadily and the number of HIV-related deaths declines, the number of OPLWH increases [5]. Both HIV infection and anti-retroviral treatment (ART) are associated with a range of chronic conditions that occur in older adults such as cardiovascular disease, diabetes, and osteoporosis, presenting new challenges for the management of these conditions [6]. OPLWH are expected to live longer in the era of ART, but at the same time, they are at risk for developing various health complications as a consequence of a life with a viral infection, exposure to medications that carry their own toxicity and side effects, and the natural effects of aging on the immune system [7]. Because senescence is an inherent process that can be accelerated by HIV, it is important to identify strategies that can modify this phenomenon.

Functional status is the ability of an individual to perform normal activities of daily living in order to meet basic needs, fulfill usual roles, and maintain health and well-being [8]. A faster rate of decline in functional status and physical performance levels have often been observed in HIV populations in comparison to their HIV-negative counterparts [9]. The rates of reduced muscle mass and strength (sarcopenia), and bone mineral density are higher among OPLWH [10]. Increased physical activity levels are recommended in order to reduce functional impairment in OPLWH [11].

In addition to the serological and physical changes often observed in HIV populations, mental and psychological disorders can also present [12]. Regular exercise has been beneficial in addressing mental health issues and psychological functioning in PLHIV [13]. Aerobic exercise has the capacity to reduce the severity of depression while a combination of aerobic and resistance exercise can decrease anxiety levels considerably in PLHIV [14, 15].

The impact of regular physical activity or exercise in HIV-negative populations have been extensively documented. Benefits include improved cardiovascular health, decreased risk of diabetes, decreased rates of osteoporosis and osteoarthritis, and improved neuropsychological health [16–18]. Together, aerobic and resistance exercise has been shown to decrease fatigue and improve quality of life, while resistance training alone has revealed a positive bearing on muscle mass and strength, functional capacity, and overall energy requirements in the elderly population [19–21]. Overall functional fitness levels in the elderly are also observed following participation in progressive, multifaceted group exercise programs [22].

From a HIV perspective, emerging data suggests that while physical activity may not have a positive impact on viral replication and on the immune system of PLHIV, it can elicit improvements in cardiorespiratory fitness, strength, body composition, and overall quality of life [23]. Aerobic exercise alone has been shown to improve pulmonary functions such as forced expiratory volume (FEV_1_), forced vital capacity (FVC), and peak expiratory flow rate (PEFR) and also alleviate respiratory and depressive symptoms in younger PLHIV [24]. A combination of aerobic and resistance training contributes positively to maintain lean body mass, cholesterol, and triglyceride indices, while reducing body fat mass and body fat percentage [25]. Cross-sectional studies also report improved neurocognitive functioning and everyday functioning in younger PLHIV who participate in regular physical activity [26–28]. Moreover, exercise for PLHIV can be regarded as safe because, whilst it does not compromise immune function, it can be beneficial in boosting functional capacity, strength, physical fitness, and mood and in ameliorating wasting and lipodystrophy [29, 30]. As people age with HIV, the prescription of physical activity should be included as part of their comprehensive medical management. General physical activity guidelines do currently exist for PLHIV [31, 32]. However, there are no clear guidelines for physical activity and exercise prescription for OPLWH, specifically those 50 years of age or older.

The objective of this scoping review is to map the existing literature and current exercise or physical activity prescriptions for OPLWH (≥ 50 years of age). It is anticipated that the results of this study will provide a consensus on the identification and mapping of key criteria and aspects of care in the context of exercise rehabilitation for OPLWH. The review will attempt to locate and report on all available studies that use exercise as a rehabilitative tool in OPLWH.

## Methodology

The present protocol has been registered within the Open Science Framework platform (registration ID: https://osf.io/728kp/). The study protocol is being reported in accordance with the reporting guidance provided in the Preferred Reporting Items for Systematic Reviews and Meta-analyses protocols (PRISMA-P) statement [33] and the PRISMA extension for scoping reviews (PRISMA-ScR) [34] (see PRISMA-P checklist in Additional file 1). It will review the literature reporting on exercise or physical activity prescriptions for OPLWH (50 years and older). This review will be guided by the methodological framework outlined by Arksey and O’Malley [35]. This framework identifies six stages that must be considered when developing a scoping review. Thus, this review will employ the following six steps accordingly: (1) identification of the research question, (2) identification of relevant studies; (3) selection of eligible studies; (4) charting the data; (5) collation, summarization, and reporting of the results; and (6) consultation with relevant stakeholders [21].

### Identification of the research question

The overall study aim underpinning this review is “What are the effects of exercise and/or physical activity prescriptions in OPLWH.”

The research questions have been defined as follows:

1. What are the physical health-related outcomes of exercise or physical activity in OPLWH?
2. What are the mental health-related outcomes of exercise and physical activity in OPLWH?
3. How does exercise or physical activity influence functional status levels in OPLWH?
4. What are the exercise recommendations for OPLWH?

### Information sources and search strategy

Identification of studies relevant to this review will be achieved through the utilization of the search strategy as recommended by the Joanna Briggs Institute [36]. A search for literature will be conducted on the following electronic databases: Google Scholar, PubMed, MEDLINE, and Cochrane Library. No date limits will be imposed on the search strategy. The Boolean terms “AND,” “OR,” and “NOT” will be used to separate keywords. A draft search strategy in PubMed is documented in Additional file 2. Further potentially relevant studies will be identified by conducting a search of the references of the included articles and searches on websites such as the World Health Organization (WHO) and the Directory of Research on Ageing. Consultation with a health librarian employed by the University of KwaZulu-Natal library services will be ongoing throughout the search process.

### Selection of eligible studies

The Population Concept Context (PPC) framework will be used to align the study selection with the research question. To be included in the review, papers will need to include adults 50 years and older living with HIV, measure or focus on specific dimensions of physical activity, and exercise in the proposed conceptual framework (e.g., quality of life, functional status, activities of daily living). They will also need to include a description of the intervention used (e.g., aerobic exercise, anaerobic exercise, strengthening, flexibility, resistance, and balance activity). Peer-reviewed journal papers will be included if they are written in English, involved human participants, and described a measure for physical, mental, or functional status of physical activity/exercise and/or the recommendations in OPLWH. Quantitative (e.g., randomized controlled trials, observational studies—cohort, case-control), qualitative, and mixed-method studies will be included in order to consider different aspects of measuring physical activity and quality of life. Papers will be excluded if they did not fit into the conceptual framework of the study or focused on a different communicable chronic condition. Commentaries or opinion pieces will be excluded.

#### Inclusion criteria

All articles or studies eligible for selection must meet the following inclusion criteria:

- Older people living with HIV (≥ 50 years of age)
- All published peer-reviewed research articles
- Articles written in English

#### Exclusion criteria

Articles or studies will be excluded if they have any of the following criteria:

- Studies where full-text articles cannot be obtained
- Commentaries or opinion pieces

Eligible articles will be uploaded into Mendeley software for Windows 10. This software program will allow for the identification and removal of all duplicated articles. Title and abstract screening of all eligible articles will be conducted by the authors. Two reviewers (VC and LC) will initially screen the citations by title and abstract to ensure that the selected studies are related to the research objectives. Excluded citations will be reviewed by a third reviewer (SC). Full-text screening of the selected articles will then be conducted by both reviewers independently (VC and LC). Every effort will be undertaken to ensure that full-text articles are obtained—these include searching of the web, engaging with the University subject librarian, or even contacting the author/s if necessary. Significant discrepancies and lack of agreement between both reviewers will be resolved through discussion. However, should there still be no resolution, a third reviewer (SC) will be employed to ensure consensus. The degree of agreement between reviewers will be calculated and reported using Cohen’s kappa coefficient.

### Charting the data

Data from eligible studies will be chartered using a standardized data abstraction tool designed for this study. The tool will capture the relevant information on key study characteristics and detailed information on all metrics used to describe the effects of physical activity and exercise and the recommendations in OPLWH. Information of interest will include the following:

- Study characteristics: study design, year of publication, journal, sample size and setting, country of origin, aim/objective of the study, and other fields to capture data relevant to the assessment of study validity.
- Participant characteristics: population sampled, age (e.g., mean with standard deviation, range), and gender (e.g., percentage of male/female participants).
- Interventions: physical activity and exercise (e.g., type and duration or intensity).
- Outcome results (e.g., findings relevant to study objectives).
- Key relevant findings and conclusions.

The final data-charting form will be jointly developed by two reviewers to determine which variables to extract (see draft of data-charting form in Additional file 3). The two reviewers will independently chart the data, discuss the results and continuously update the data-charting form in an iterative process. Any disagreements will be resolved through discussion between the two reviewers or further adjudication by a third reviewer.

### Collation, summarization, and reporting of the results

A scoping review is often used to map the concepts underpinning a research area as well as the main sources and types of evidence that are available. The findings gathered in this review protocol will provide an overview of the research together with an assessment of the quality of individual studies. The authors will produce a narrative report which will summarize the extracted data around the following outcomes: region of study, physical activity outcomes in HIV, and exercise rehabilitation prescription for OPLWH. The results will be described in relation to the research question and sub-questions in the context of the overall study purpose. It will also be depicted in the final write-up using a thematic approach that allows for flexibility in the capturing of data, because it may be possible for the review to yield unknown concepts. Gap identification will detect areas, such as countries that lack data on rehabilitation outcomes for OPLWH.

### Quality appraisal

The quality of the studies selected from the search strategy will be appraised using the Mixed Methods Appraisal Tool (MMAT) version 2018 [37]. Categories within the MMAT allows for the inclusion of qualitative studies, category 2 quantitative randomized control trial, category 3 non-randomized trials, category 4 quantitative descriptive studies, and category 5 mixed methodologies studies. Three reviewers (LC, VC, and SC) will be involved in the quality appraisal process. Two reviewers (LC, SC) will capture the methodological quality of the selected studies as per the criteria outlined in the MMAT, while a third reviewer (VC), who has vast experience in the application of MMAT in the quality appraisal process, will oversee the process. In order to enhance the methodology of a scoping study, Daudt et al. 2013 strongly suggest assessing the quality of studies and conducting a trial of the method before fully embarking on the charting process in order to ensure consistency [38].

## Discussion

The proposed scoping review will aim to identify and describe the effects of physical activity or exercise prescription in OPLWH. It will also highlight gaps regarding the use of physical activity as an effective rehabilitation tool in OPLWH. This review will be the first part of a study that aims to develop physical activity guidelines for OPLWH in sub-Saharan Africa. An understanding of the physical activity outcomes will assist health professionals to confidently design and implement physical activity programs that best address the needs and goals of OPLWH. This will capacitate health professionals to provide suitable care for OPLWH at both primary and secondary care levels. This review also has the potential to create greater awareness of the growing health care needs of OPLWH and will provide evidence to assist stakeholders and health policymakers to address the needs of this vulnerable population. A potential limitation of our approach to the scoping review would be a language bias as research evidence in English will be considered. Although the researchers do not anticipate protocol amendments, issues that arise with the original protocol will be documented in the review paper under the methodology section.

## Supplementary information

**Additional file 1.** PRISMA 2009 Checklist.

**Additional file 2.** Draft Search Strategy.

**Additional file 3.** Data Charting form.

## Data Availability

Not applicable

## Abbreviations

ARV: Anti-retroviral treatment
FEV_1_: Forced expiratory volume
FVC: Forced vital capacity
HIV: Human immunodeficiency virus
MMAT: Mixed Methods Appraisal Tool
OPLWH: Older people living with human immunodeficiency virus
PEFR: Peak expiratory flow rate
PLHIV: People living with human immunodeficiency virus
PPC: Population Concept Context
