# Supplementary material for: Physical activity and exercise for older people living with HIV: a protocol for a scoping review
Source: Syst Rev. 2020 Mar 20;9:60. doi: 10.1186/s13643-020-01327-4 (PMC7085181; doi:10.1186/s13643-020-01327-4)
Supplement: Supplementary file 2 — Additional file 2. Draft Search Strategy. [file 13643_2020_1327_MOESM2_ESM.pdf]

DRAFT SEARCH STRATEGY

Keywords Searched	Data Base
("anthropometric" OR "quality of life" OR "BMI" OR "Body Mass Index" OR "WHR" OR "Waist hip ratio" OR "skin fold" OR "functional status" OR "function" OR "ADL*" OR "activity* of daily living" OR "heart rate" OR "blood pressure") AND (("physical activity" OR "exercise" OR "exercise rehabilitation" OR "Exercise intervention" OR "aerobic exercise" OR "Anaerobic exercise" OR "combination exercise" OR "strength exercise" OR "flexibility" OR "agility") AND (("geriatrics" OR "aged" OR "older" OR "ageing" OR "senescence" OR "old") AND ("HIV" OR "Human immunodeficiency virus" OR "AIDS" OR "acquired immune deficiency syndrome")))	PubMed
