# Supplementary material for: Physical activity and exercise for older people living with HIV: a protocol for a scoping review
Source: Syst Rev. 2020 Mar 20;9:60. doi: 10.1186/s13643-020-01327-4 (PMC7085181; doi:10.1186/s13643-020-01327-4)
Supplement: Supplementary file 3 — Additional file 3. Data Charting form. [file 13643_2020_1327_MOESM3_ESM.pdf]

DATA CHARTING FORM

Title of Study:	
Author(s)	
Year of Publication	
Country of Origin (where the study was conducted)	
Aim/Objective of Study	
Study Setting	
Study Population	
Participants Characteristics (Gender, CD4 Count, % on ARV, % Undetectable, % Adherent)	
Sampling (method and size)	
Study Design	
Physical activity (type or duration or intensity)	
Data Analysis	
Conclusion	
Outcomes (findings relevant to study objectives)	
Key Relevant Findings	
Conclusion	
